# Validation of the face-name pairs task in major depression: impaired recall but not recognition

**DOI:** 10.3389/fpsyg.2014.00092

**Published:** 2014-02-12

**Authors:** Kimberley J. Smith, Sinead Mullally, Declan McLoughlin, Shane O’Mara

**Affiliations:** ^1^Trinity College Institute of Neuroscience, Trinity College DublinDublin, Ireland; ^2^Neurobiology of Depression Research Group, St. Patrick’s HospitalDublin, Ireland

**Keywords:** depression, recall, recognition, cognition, memory, face-name pairs

## Abstract

Major depression can be associated with neurocognitive deficits which are believed in part to be related to medial temporal lobe pathology. The purpose of this study was to investigate this impairment using a hippocampal-dependent neuropsychological task. The face-name pairs task was used to assess associative memory functioning in 19 patients with major depression. When compared to age-sex-and-education matched controls, patients with depression showed impaired learning, delayed cued-recall, and delayed free-recall. However, they also showed preserved recognition of the verbal and nonverbal components of this task. Results indicate that the face-name pairs task is sensitive to neurocognitive deficits in major depression.

## INTRODUCTION

Major depressive disorder (MDD) is one of the leading causes of disability worldwide, with an estimated 350 million people affected (World Health Organisation, 2012). Some of the most occupationally debilitating symptoms of depression are the widespread neurocognitive deficits (Mackin and Arean, 2009; Baune et al., 2010) that often accompany the core pathological symptoms of depressed mood and anhedonia (DSM-IV-TR, 2004). These cognitive impairments include deficits in working memory, attention, verbal fluency, executive functioning, and long-term memory (Landro et al., 2001; Rogers et al., 2004; Baune et al., 2010; Hammar and Ardal, 2009; Reppermund et al., 2009).

Long-term memory impairments are in part characterized by poorer delayed recall of both verbal and nonverbal items (Reischies and Neu, 2000; Landro et al., 2001; Naismith et al., 2003; Vythilingam et al., 2004). As well as showing poorer recall, some studies also demonstrate depressed patients to have impaired recognition with preserved familiarity (MacQueen et al., 2002; Ramponi et al., 2004; Drakeford et al., 2010). This memory impairment is believed to be the result of hippocampal pathology as depressed patients show reduced hippocampal volumes (Sheline et al., 1996, 1999; Mervaala et al., 2000; Bremner et al., 2004; Frodl et al., 2006) that can be correlated with the degree of memory impairment (Hickie et al., 2005).

The face-name pairs task (FNP) is a widely regarded paradigm of associative memory which is associated with activation of the anterior hippocampus (Sperling et al., 2003; Zeineh et al., 2003; Chua et al., 2007). This task has been previously used and shown to have sensitivity in various clinical and psychiatric populations including people with bipolar disorder (Glahn et al., 2010) and Alzheimer’s disease (van Paasschen et al., 2013). However to our knowledge, this test has never been used in patients with a MDD. The aim of this study was to assess whether the face-name pairs task was sensitive to 1. Differences in recall and recognition in a depressed population and 2. Differences between a Depressed group and a Control group.

## METHODS

Nineteen in-patients MDD were recruited from St. Patrick’s Hospital Dublin and compared to 17 age-education-and-sex-matched control participants.

Depressed patients were identified with the help of nursing staff, and by assessing patient charts for current psychiatric diagnosis Potential participants were then approached directly to ask whether they were willing to take part. Inclusion criteria included being between the ages of 18–65 and having MDD of mild to moderate severity (as indicated by a severity of ≥8 on the Hamilton Depression Inventory). Presence of current MDD (past month) was confirmed by completion of the Structured Clinical Interview for DSM-IV disorders prior to study commencement. Exclusion criteria included any other significant medical or psychiatric co-morbidity, or being on medication such as antipsychotics or which could adversely influence results (see **Table 1** for demographic information).

**Table 1:** Table showing demographic information for the participants in this study.

Group	*N*	Age	Gender	Education
Control	17	46.24 ± 3.2	Male: 11 (65%); female: 6 (35%)	14.5 ± 0.5
Depressed	19	39.32 ± 2.8	Male: 13 (68%); female: 6 (32%)	13.3 ± 0.4

*There was no significant difference between the groups when assessed using an independent-samples *t*-test for age [*t*(34) = 0.52, *P* = 0.1], or education [*t*(34) = 0.93, *P* = 0.4]. When gender was assessed using a chi-squared analysis, there was no significant difference between groups for the percentages of males and females [*x^2^*(1, *N = *35) = 0.23, *P* = 0.6].*

Control participants were recruited via the Trinity College Dublin electronic notice board and an advertisement in the Irish Times newspaper. Inclusion criteria included being between 18 and 65 years of age, and exclusion criteria included having any significant medical co-morbidities, being pregnant, or current psychiatric morbidity (as confirmed by completion of the structured clinical interview). This study was carried out with ethical approval from the psychology ethics counsel of Trinity College Dublin and the ethics board at St. Patrick’s Hospital. All participants gave their full informed consent prior to the commencement of testing.

## MATERIALS AND METHODS

Participants were tested using the 28-item Everyday Memory Questionnaire (EMQ) and a computerized version of the FNP task as described below.

### FACE-NAME PAIRS TASK

#### Materials and stimuli

**FIGURE 1:**
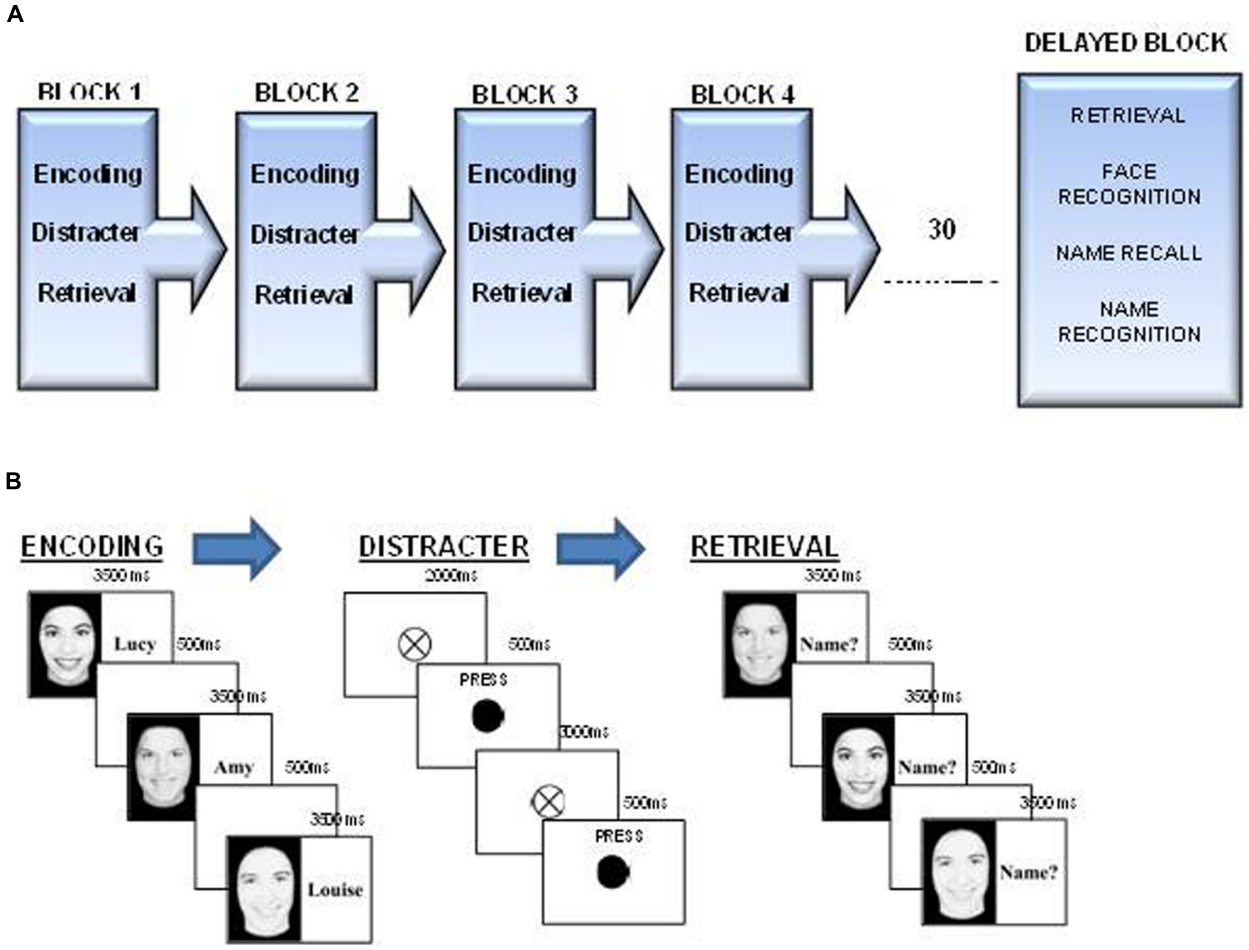
**(A)** FNP running order. The Face-Name task comprises four blocks of encoding, distraction, and immediate retrieval followed by a block of delayed retrieval, which also involved name recall and face and name recognition components. **(B)** FNP; Encoding, distracter, and retrieval components. Participants first encoded the six faces and name for 3500 ms each, following this they carried out a distracter task where they were required to press the spacebar every time the fixation cross turned black. Each block ended with the retrieval component where they were asked to call out the name they believed belonged to each face.

This face-name task is a modified version of the design described by Zeineh et al. (2003). In order to avoid potential problems with “floor” results, due to the older age of the population studied, the number of faces to be remembered was reduced from eight to six. The six faces presented were all female (selected from a college yearbook, all presented in black and white and with all their hair removed). During both the encoding and retrieval phases these faces were presented on the right half of the screen. In the encoding phase the left side of the screen would contain the corresponding name, and in the retrieval phase the name was replaced by the prompt “Name?” (see **Figure 1**).

#### Procedure

This task comprised four blocks of immediate recall, where participants were presented with six face-name pairs (see **Figure 1B**) followed by a distracter task (see **Figure 1B**) and finally retrieval, where participants were prompted to vocally recall the name of each face (see **Figure 1B**). Following a half hour break one block of delayed recall with face and name recognition components where participants were required to identify faces and names that were and were not previously seen (see **Figure 1A**). More details on each phase of the task are included below;

***Face name encoding.*** During the encoding blocks, participants viewed six face-name pairs (each face was viewed once per block), which were presented serially at a rate of 3.5 s per pair, with an inter-stimulus interval of 500 ms. The presentation order was constant across each of the encoding blocks.

***Distracter Task.*** Between each encoding and retrieval block, a distracter task was presented to participants for 35 s. During this task participants saw a fixation cross (20 mm) presented in the center of the screen, and at pseudo-random intervals of between 2 and 4 s the circle would turn black for 500 ms. Participants were instructed to press the space-bar whenever the circle “flashed” black as quickly and accurately as they could. A total of 10 targets were presented to the participants.

***Immediate face name retrieval.*** Following the distracter task participants viewed the six faces, which were presented in a randomized order, this time without the accompanying names. Each face was presented for 3.5 s, with an inter-stimulus interval of 500 ms, upon presentation of each face, participants were asked to vocally recall the name that they believed to correspond to each face. The experimenter recorded correct and incorrect responses, with a non-response being recorded as incorrect.

#### Delayed face name retrieval

After a 30-min delay, where participants were completing other tasks, participants were required to complete the recall as many correct face-name pairs as possible.

***Delayed name recall.*** Following the 30 min delay, participants were asked to vocally recall as many names as they could remember from the experiment, the total names correctly recalled was summed and reported.

***Delayed face and name recognition.*** Following the delayed Name Recall and delayed Face Name Retrieval, participants were instructed that they would be see fourteen faces on the screen, presented centrally. Each face remained on the screen until the participant chose to press the spacebar to move onto the next face. Six of the faces were those that had been seen in the experiment and the other eight were faces that had not previously been seen. For each face participants were instructed to circle “yes” or “no” on their response sheet, and then rate their certainty of their response by circling a number between 1 and 6, where 1 was representative of them being highly confident of their response, and 6 being very unconfident of their response. They were then required to complete the same process for names. The total accuracy for this part of the experiment was the number of faces which were part of the experiment which were correctly identified as being part of the experiment. Misses were calculated as those faces which were part of the experiment, but were not identified by the participant. Correct foils were calculated as the total number of faces which were correctly identified as not being part of the experiment. False positives were calculated as those faces which were incorrectly identified as being part of the experiment.

The total certainty of responses was summed by calculating the percentage certainty for each response, and then adding together each of the fourteen percentage’s together to create a total score.

***Everyday memory questionnaire.*** This self-rating questionnaire (Sunderland et al., 1984) consists of twenty-eight questions which measure how often within the last 3 months participants believe they have had problems with aspects of memory which people should encounter on an everyday basis. The relative frequency of perceived memory problems are rated on a nine-point scale which ranges from “Not at all in the last 3 months” to “More than once a day,” with higher scores indicative of more perceived problems with memory.

The total score was obtained by summing the individual scores (from 0 to 8 per question). The square root of the total score was then calculated (as in Sunderland et al., 1984), and is the score reported in this thesis.

### STATISTICS

Results for this study were calculated using both repeated-measures and one-way ANOVA, with Bonferroni corrected *post hoc* comparisons being run where appropriate. Where the two groups were compared directly for one condition an independent-groups *t*-test was run, with adjusted degrees of freedom where appropriate.

## RESULTS

When assessed using the EMQ Depressed patients rated themselves as having significantly more everyday memory problems than matched controls [*t*(34) = 3.71, *p* < 0.01; Control 5.47 ± 0.5; Depressed 8.08 ± 0.5].

### RECALL PERFORMANCE

**FIGURE 2:**
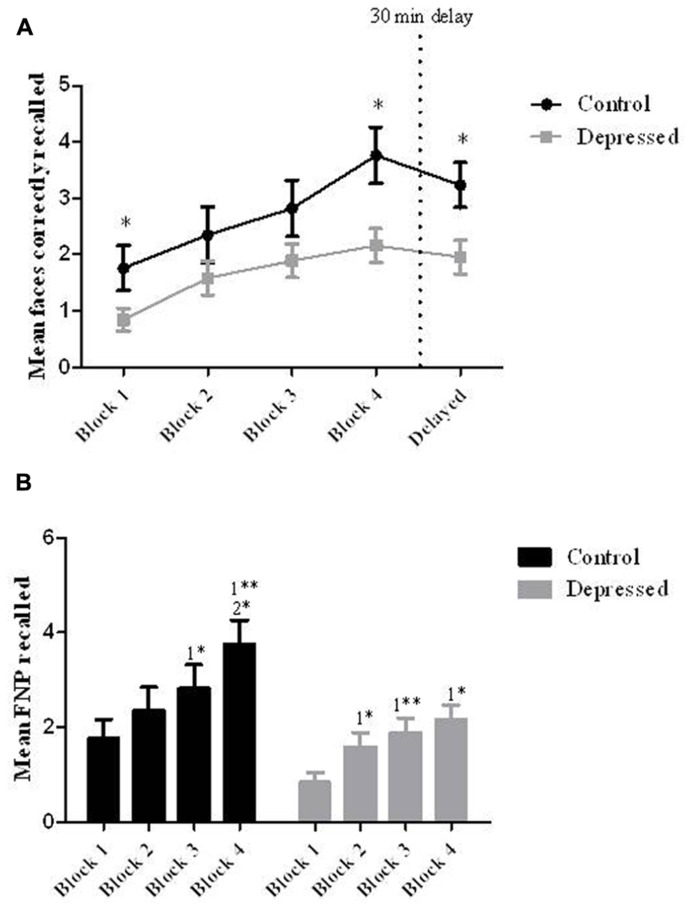
**(A)** Recall between-groups. When compared to controls patients with depression showed impaired learning for the six FNP at learning blocks 1 and 4, as well as showing an impairment at the delayed cued-recall of FNP (*p < 0.05). **(B)** Learning within-groups. When assessed for learning with-groups the control group performed significantly better in the last two blocks relative to the first two blocks (*1 relative to block 1; *2 relative to block 2), and the depressed group performed significantly better in blocks 2–4 relative to the first block (*p < 0.05, **p < 0.01). Error bars represent SEM.

When performance on the FNP test was measured between-groups using a one-way ANOVA, there was a significant effect of group at learning block 1 [*F*(1,35) = 5.03, *p* < 0.05], block 4 [*F*(1,35) = 7.19, *p* < 0.05] and the delayed recall block [*F*(1,35) = 6.66, *p* < 0.05; see **Figure 2A**].

When groups were assessed individually using a repeated-measures ANOVA, there was an overall effect of block on learning for the Control group [*F*(4,64) = 9.12, *p* < 0.01]. *Post hoc* tests showed a significant improvement in performance from block 1 (*M *= 1.77, SD**= 0.4: 29.5%) to block 4 (*M *= 3.76, SD**= 0.5: 62.7%) and also from block 1 to the delayed block (*M *= 3.24, SD**= 0.4: 54.0%; see **Figure 2B**).

When the Depressed group was assessed there was also a significant effect of block on performance [*F*(4,72) = 5.67, *p* < 0.01]. *Post hoc* tests showed that all significance was relative to performance in block 1 (*M *= 0.84, SD**= 0.2: 14%) with improvements from this block been seen at block 2 (*M *= 1.58, SD**= 0.3: 26.3%) block 3 (*M *= 1.9, SD**= 0.3: 31.7%) block 4 (*M *= 2.16, SD**= 0.3: 36.0%) and the delayed block (*M *= 1.95, SD = 0.3: 32.5%; see **Figure 2B**).

**FIGURE 3:**
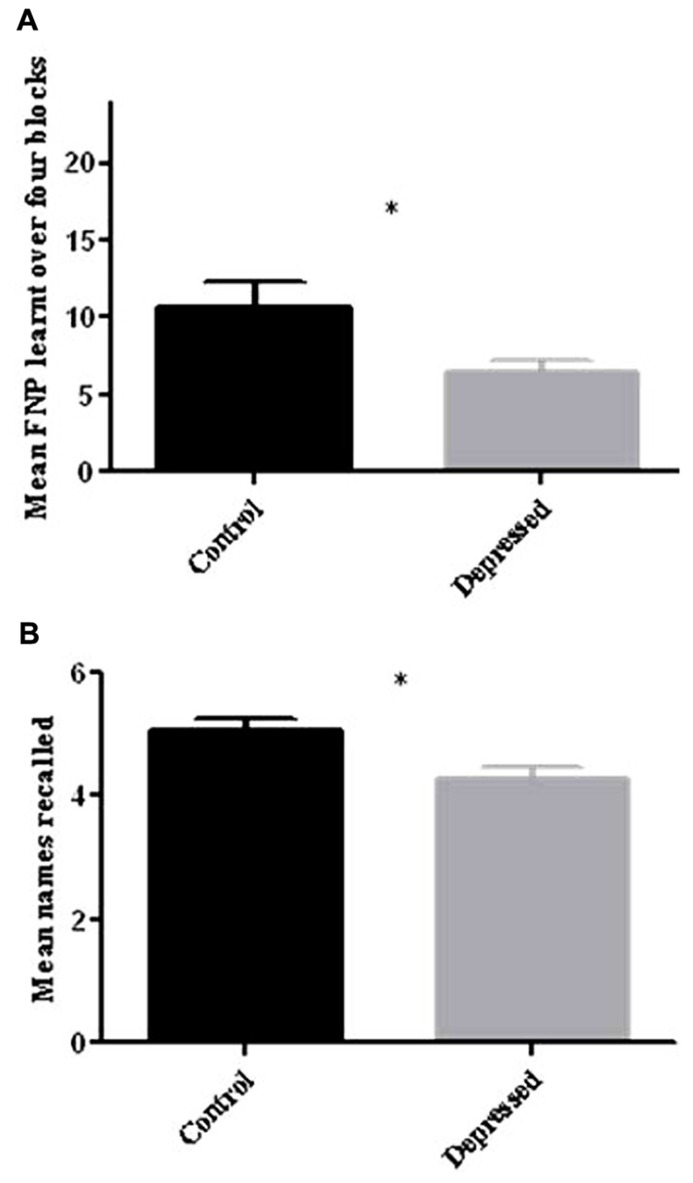
**(A)** Total FNP recalled over four learning blocks. When compared to control participants depressed patients recalled significantly fewer FNP over the four learning blocks (*p < 0.05). **(B)** Free recall of names following half hour delay. When compared to control participants depressed patients recalled significantly fewer names after the half hour delay (*p < 0.05). Error bars represent SEM.

When assessed for total learning over the first four blocks using an independent-samples *t*-test, there was a significant difference between the two groups [*t*(25.6) = 2.35, *p* < 0.05], with the Depressed group recalling fewer faces than the Control group (Control: 10.71 ± 1.61: 44.6%; Depressed: 6.47 ± 0.8: 30.0%; see **Figure 3A**).

When the two groups were compared for delayed free recall of names for the task, there was a significant effect of group [*t*(34) = 2.43, *p* < 0.05), with the Depressed group recalling significantly fewer names (Control: 5.06 ± 0.2: 84.3%; Depressed: 4.26 ± 0.2: 71.0%; see **Figure 3B**).

### RECOGNITION PERFORMANCE

**FIGURE 4:**
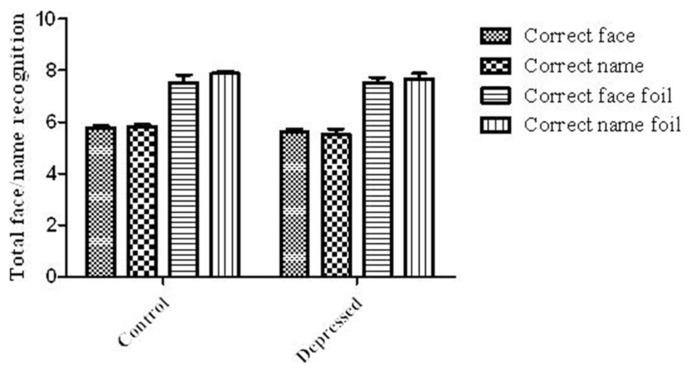
**Recognition of faces and names.** There was no significant difference between the two groups for lures and foils for both the faces and names. Error bars represent SEM.

Despite there being a significant impact of Depression on the recall portion of the FNP task, there was no significant impact of Depression on any of the measures of recognition (see **Figure 4**), including the correct number of faces identified [*t*(34) = 0.85, *P* = 0.4; Control: 5.76 ± 0.1: 96.0%; Depressed: 5.63 ± 0.1: 93.8%] or correct face foils identified [*t*(34) = 0.008, *P* = 1; Control: 7.53 ± 0.3: 94.1%; Depressed: 7.53 ± 0.2: 94.1%]. There was also no effect on the number of names correctly recognized [*t*(26.1) = 1.38, *P* = 0.2; Control: 5.82 ± 0.1: 97.0%; Depressed: 5.53 ± 0.2: 92.2%], or correct foils identified [*t*(26.9) = 1.14, *P* = 0.3; Control: 7.88 ± 0.08: 98.5%; Depressed: 7.68 ± 0.2: 96.0%].

## DISCUSSION

Compared to age-sex and -education matched healthy controls depressed patients showed impairment in aspects of the FNP task related to associative learning, delayed cued-recall and delayed free-recall of names. This indicates that the face-name pairs task is sensitive to mneumonic impairment in a depressed patient-population.

When assessed within-groups over the four learning blocks, there was a significant increase in FNP recall relative to block 1 indicating that the Depressed group was learning over the course of the task. These responses however were at a significantly lower level than the Control group. This finding is supported by previous studies that show decreased performance of depressed patients in tasks such as the California verbal learning task (CVLT). This is believed to be due to impairment at the learning phase of the task (Elderkin-Thompson et al., 2006, 2007) as reflected by effects on immediate recall and the total rate of learning (Kizilbash et al., 2002). Other studies have also found that depressed patients are impaired in the early stages of memory tasks when encoding demands are high as well as in the delayed recall portion of a task (Brand et al., 1992). These studies support observations from this experiment where depressed patients showed a significant impairment at block 1 of the task as well as the delayed block. The impairment seen was specific to the recall and encoding portions of the experiment but not the recognition portion of the experiment, suggesting that Depressed patients are impaired in verbal-recall and associative-recall, but not recognition. This observation is supported by few studies (Calev and Erwin, 1985), with the majority of literature in this area finding that both recall and recognition are adversely affected by depression (MacQueen et al., 2002; Ramponi et al., 2004; Drakeford et al., 2010). However, this is possibly due to low number of target items and foils participants were required to identify. For example, Drakeford et al. (2010) found that depressed subjects showed impaired recollection but intact familiarity when subjects were required to identify 50 targets from a total of 100 items, with many other remember/know paradigms also using a large number of lures and foils (MacQueen et al., 2002).

However, it must also be acknowledged that alternative explanations also exist for the observed deficit in people with MDD. Both motivational and attentional deficits comprise some of the core symptoms of MDD, and it is possible that deficits in attention and/or motivation could be responsible for worsened recall but intact recognition (as recognition is less cognitively demanding). Some researchers hypothesize that problems with directing attention (Li et al., 2013) and task motivation (Scheurich et al., 2008) could be in part responsible for poorer performance in memory tasks within this population.

Results from this study should also be interpreted with caution due to the small sample size and various potentially biasing confounders which were not explicitly controlled for or investigated. Factors that could have an influence on cognitive performance in this population that were not explicitly investigated in this study include age, consumption of nicotine and the impact of potential sleep deprivation. Another important issue that could not be investigated or controlled for due to the small sample was examination of the subtype of depression, depression severity and length of depressive episode. However, it should be noted that participants in this study were hospitalized for their depression indicating that the depressive episode was significantly disabling. However, reviews of the neuropsychological functioning of depressed patients describe variable results regarding cognitive functioning in this population, and present evidence that depression severity is not necessarily related to performance (McClintock et al., 2010). Some researchers also suggest that the cognitive impairment in depression is non-specific and that the pathological symptoms of depression can be dissociated from neurocognitive symptoms (Reppermund et al., 2009).

Further methodological limitations of the study include the use of only female faces only in the task and the fact that there were not enough control participants to complete perfect matching with Depressed patients. However, there was no evidence from this study that there was a gender bias implicit in the task. Strengths of this study include the fact that, to our knowledge, this is the first investigation of the FNP task in people with depression. The FNP also has high ecological validity when compared to other memory tasks due to the use of faces and names.

While results from this task would suggest hippocampal dysfunction in depressed patients a functional imaging study needs to be conducted in this cohort using this task in order to elucidate the precise neuroanatomical structures involved in this deficit.

## CONCLUSION

In conclusion there is evidence that Depressed patients do show learning over repeated blocks of the FNP associative memory task, but that this is at a significantly lower level than age-sex-and-education matched Control participants. The depressed cohort studies here also showed impaired delayed recall (associative and verbal), however, there was a preservation of verbal and nonverbal recollection which is proposed to be due to the low number of targets and foils for this task.

## Conflict of Interest Statement

The authors declare that the research was conducted in the absence of any commercial or financial relationships that could be construed as a potential conflict of interest.
